# Survival Determinants and Treatment Outcomes of Patients with Small Cell Lung Cancer and Brain Metastases: A U.S. National Analysis †

**DOI:** 10.3390/cancers17233833

**Published:** 2025-11-29

**Authors:** Khalid Ahmad Qidwai, Zouina Sarfraz, Khalis Mustafayev, Lydia C. Hodgson, Arun Maharaj, Triparna Sen, Tulika Ranjan, Manmeet S. Ahluwalia

**Affiliations:** 1 Department of Medical Oncology, Miami Cancer Institute, Baptist Health South Florida, Miami, FL 33176, USA; khalid.qidwai@baptisthealth.net (K.A.Q.); zouina.sarfraz@baptisthealth.net (Z.S.);; 2 Department of Internal Medicine, The Ohio State University, Columbus, OH 43210, USA; 3 The Ohio State University Comprehensive Cancer Center—Arthur G. James Cancer Hospital and Richard J. Solove Research Institute, Columbus, OH 43210, USA; 4 Dartmouth Hitchcock Medical Center and Clinics, Lebanon, NH 03756, USA; 5 Herbert Wertheim College of Medicine, Florida International University, Miami, FL 33199, USA

**Keywords:** small cell lung cancer, brain metastases, stereotactic radiosurgery, systemic therapy, overall survival, treatment patterns, National Cancer Database

## Abstract

**Background/Objectives**: Brain metastases (BM) are common in small cell lung cancer (SCLC) and portend poor outcomes. Contemporary determinants of survival in the modern treatment era remain incompletely defined. We evaluated factors associated with overall survival (OS) among patients with SCLC and BM using a recent, nationally representative dataset. **Methods**: We identified adults diagnosed with SCLC and brain metastases between 2018 and 2020 in the National Cancer Database (NCDB). Demographic, clinical, treatment, and survival data were extracted for analysis. Unadjusted OS was estimated using Kaplan–Meier methods. Multivariable Cox proportional hazards models identified factors associated with mortality, with proportional hazards (PH) assessed using scaled Schoenfeld residuals. Complementary Accelerated Failure Time (AFT) modeling was performed to confirm robustness. **Results**: Of 62,671 SCLC cases, 11,074 (17.7%) had BM, including 32.6% with brain-only disease. Median overall survival (mOS) was 6.6 months (95% CI, 6.47–6.87); patients with brain-only disease had an mOS of 8.8 months (8.38–9.26), compared with 5.95 months (5.75–6.18) for those with concurrent extracranial metastases. In multivariable analysis, age ≥ 65 years (HR 1.13, *p* < 0.001) was associated with higher mortality, whereas female sex (HR 0.87, *p* < 0.001), Black (HR 0.88, *p* = 0.001), Asian (HR 0.80, *p* = 0.022), and Hispanic (HR 0.87, *p* = 0.008) patients had lower hazards. Worse outcomes were associated with public or no insurance, lower income, non-academic facilities, and extracranial metastases; educational attainment was not significant. Proportional hazards assumptions were largely met with minor deviations, and AFT modeling confirmed consistent results. Treatment modality remained independently associated with survival in both models. **Conclusions**: In this contemporary national cohort, survival among patients with SCLC and brain metastases was influenced by multiple clinical, sociodemographic, and treatment factors, including age, sex, insurance status, facility type, and extent of metastatic disease. Treatment modality remained an independent predictor of survival. These results provide updated real-world benchmarks and highlight the need for prospective studies to define optimal management strategies in this high-risk population.

## Simple Summary

Brain metastases are common in small cell lung cancer and often lead to poor survival. Using data from over 11,000 patients across the United States, we examined how various determinants influence survival outcomes. We found that treatment choice affected prognosis. Patients who received stereotactic radiosurgery with systemic therapy lived the longest, followed by those treated with whole-brain radiotherapy plus systemic therapy. Whereas patients who received only one treatment modality or no therapy had significantly shorter survival. Younger age, female sex, higher income, private insurance, and treatment at academic centers were also linked with better outcomes. Black and Asian patients had improved survival compared to White patients. Both access to care and treatment decisions play major roles in survival, highlighting the need for equitable delivery of therapies.

## 1. Introduction

In 2025, an estimated 226,650 new cases of lung and bronchus cancer are expected, accounting for approximately 11% of cancer diagnoses in the United States (US) [1]. Lung cancer is the second most common cancer worldwide and is the leading cause of cancer-related mortality, with 20.2% of all cancer-related deaths linked to lung cancer, with a 5-year relative survival of 28.1% [2,3]. Small cell lung cancer (SCLC) represents 10–15% of all diagnosed lung cancers and is associated with worse outcomes than non-small cell lung cancer (NSCLC) [4,5].

SCLC is characterized by a high proliferative rate, a significant predisposition for early metastasis, and a poor prognosis [6], and recent data highlight that both primary and acquired resistance to immunotherapy substantially contribute to its poor clinical outcomes [7,8]. SCLC is known to spread to many sites, including the liver, bones, adrenal glands, lymph nodes, and brain [9,10]. Patients diagnosed with SCLC have a two-fold higher risk of developing brain metastases (BM) compared to those with NSCLC [6]. SCLC cells’ high propensity to metastasize to the central nervous system (CNS) is noteworthy, with 10% of patients already having BM at their first clinical visit [10], and up to 80% of SCLC patients experience BM throughout their disease course, including a considerable proportion of occult cases [6]. Systemic therapies, including immune checkpoint inhibitors and targeted agents, have shown promise in improving clinical outcomes by addressing both intracranial and extracranial disease. Prophylactic cranial irradiation (PCI) remains a consideration for select patients with extensive-stage SCLC who respond well to systemic therapy (*Sys*), aiming to reduce the risk of BM [11].

Despite improvements in cancer survival rates, differences in survival and treatment outcomes persist across demographic and clinical groups. Previous studies have demonstrated that factors such as age, sex, race, insurance status, and tumor stage may influence cancer incidence and survival [12]. Studies have documented significant differences in socioeconomic factors in lung cancer and BM at diagnosis, which are reflected in oncological outcomes [13,14,15]. Some analyses suggest that survival outcomes are significantly influenced by clinical and treatment-related factors, including age, comorbidities, disease stage, and therapeutic approach [16,17]. However, most of these studies were limited to NSCLC.

Past research has shown associations between treatment and socioeconomic factors and survival outcomes in both limited- and extensive-stage SCLC [18,19]. However, little is known about how these determinants affect patients who develop BM, a subgroup with particularly poor outcomes. Understanding survival patterns in this population is especially important in the contemporary treatment era. Therefore, this study aims to ascertain how patient and treatment characteristics influence survival outcomes among individuals with SCLC and BM.

## 2. Materials and Methods

### 2.1. Data Source and Study Design

This was a retrospective cohort study that was exempt from institutional review board (IRB) approval, and the requirement for written informed consent was waived due to the use of de-identified data. The study was conducted in accordance with the Strengthening the Reporting of Observational Studies in Epidemiology (STROBE) guidelines. Data were obtained from the National Cancer Database (NCDB), a hospital-based database with data on over 70% of newly diagnosed malignancies in the US [20].

The study period began in 2018 to align with the implementation of AJCC 8th edition staging and the introduction of standardized metastasis coding across Commission on Cancer-accredited facilities under the STORE 2018 guidelines [21]. This ensured consistency in staging and improved completeness of BM data fields. Additionally, starting from 2018 allowed for accurate capture of contemporary radiation therapy modalities, including stereotactic radiosurgery (SRS) and whole-brain radiotherapy (WBRT), reflecting updates in NCDB treatment coding practices. This timeframe also captures the transition into the modern therapeutic era, coinciding with the initial clinical uptake of immunotherapy in extensive-stage SCLC [22]. This project was registered in the open science framework: https://osf.io/pqe37 (accessed on 13 October 2025). Prior presentation was performed at the ASCO Annual Meeting 2024 [23].

### 2.2. Variables and Definitions

Overall survival (OS) was defined as months from initial diagnosis to death or last contact; living patients were censored at last contact. Age at diagnosis was analyzed as <65 vs. ≥65 years; sex as male vs. female; race as White, Black, Asian, or Other; ethnicity as Hispanic vs. non-Hispanic; and insurance at diagnosis as private, Medicare, Medicaid, other government, or uninsured. Area-level median household income and educational attainment were derived by linking patient ZIP codes to American Community Survey 2016–2020 estimates [24] and were analyzed using the following cutpoints: income ≥$57,856 vs. <$57,856 and education ≥9.1% vs. <9.1% adults ≥25 years without a high-school diploma. Comorbidity burden used the Charlson–Deyo index (0, 1, 2, ≥3). Facility type was classified as academic/research, integrated network, or community. Great-circle distance (“crow-fly”) from residence to treating facility was analyzed as <11.2 vs. ≥11.2 miles. Treatment categories were mutually exclusive: SRS+Sys, WBRT+Sys, Sys only, SRS only, WBRT only, or no treatment. Metastatic pattern at diagnosis was categorized as brain metastases only vs. brain metastases with other concurrent metastatic sites (bone, liver, lung, distant lymph nodes, or other distant sites).

### 2.3. Statistical Analysis

Descriptive statistics were used to summarize baseline demographic and clinical characteristics. Categorical variables were expressed as frequencies and percentages, and continuous variables as medians with interquartile ranges (IQR). Group differences were assessed using the chi-square test for categorical variables and the Wilcoxon rank-sum test for continuous variables. Unadjusted OS was estimated using the Kaplan–Meier method and compared with the log-rank test.

Cox proportional hazards regression models were applied to evaluate the association of demographic, clinical, and treatment-related factors with OS. Hazard ratios (HR) and 95% confidence intervals (CI) were reported for both univariable and multivariable models. Covariates included in multivariable analyses were age, sex, race, ethnicity, comorbidity score, insurance type, median household income, educational attainment, treatment facility type, and treatment modality. For continuous variables, Spearman correlation *p*-values were reported; for categorical variables, chi-square *p*-values were provided.

The proportional hazards (PH) assumption was evaluated using scaled Schoenfeld residuals for each covariate. To account for potential time-dependent effects, a complementary Accelerated Failure Time (AFT) model was also fitted, providing a parametric assessment of covariate effects on survival duration.

All statistical tests were two-sided, and *p* < 0.05 was considered statistically significant. Analyses were performed using R v4.3.3 (R Foundation for Statistical Computing, Vienna, Austria).

## 3. Results

**Figure 1:**
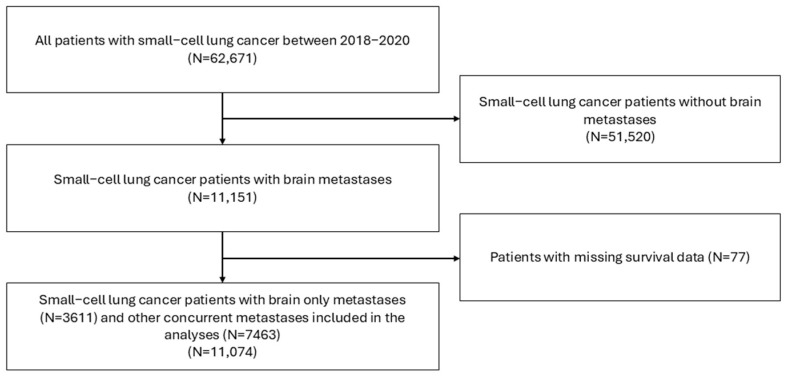
Flowchart of patient selection.

The number of reported SCLC patients in the NCDB between 2018 and 2020 was 62,671. The cohort sample size of those with SCLC BM was 11,074, of whom 32.6% (N = 3611) of patients lacked other concurrent metastases. The flowchart is given in Figure 1.

### 3.1. Patient Demographics and Baseline Characteristics

The median follow-up, defined as the time from diagnosis of SCLC-BM to last known contact or death, was 34.2 months (IQR: 24.6 to 44.4).

The median patient age was 66 years (IQR: 60–73), with 56.6% of patients 65 years or older (N = 6269). Just over half of the cohort (50.5%, N = 5591) was male, the majority was White (N = 9727, 87.8%), followed by Black (N = 1005, 9.1%), Asian (N = 147, 1.3%), and Other (N = 195, 1.8%). Most patients were non-Hispanic (10,591 patients; 95.6%), while Hispanic patients formed 4.4% of the cohort (N = 483). Of the total cohort, 57.1% (N = 6318) were Medicare insured, 42.6% (N = 4720) resided in ZIP codes with a median household income ≥$57,856, and 47.8% (N = 5291) lived in areas where ≥9.1% of adults had not completed high school. Nearly half of patients (48.6%, N = 5384) were treated at community cancer centers, and 40.5% (N = 4488) received WBRT and Sys.

Patient demographic and treatment are summarized in Table 1.

### 3.2. Survival Outcomes

**Figure 2:**
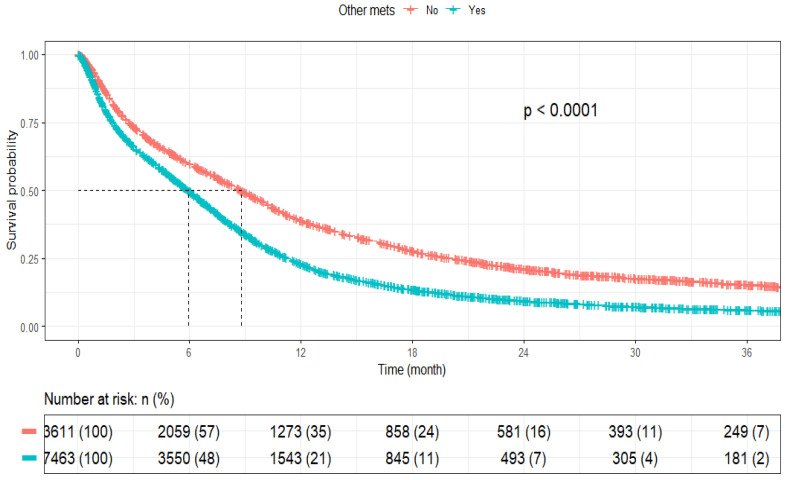
Kaplan Meier curves for patients with SCLC with or without other concurrent metastases.

The median OS (mOS) for all patients with SCLC BM was 6.6 months (95% CI: 6.47–6.87). The 3-month, 6-month, 1-year, 2-year, and 3-year survival rates were 68.6%, 53.1%, 28.2%, 13.4%, and 9.2%, respectively. SCLC patients with only BM had an mOS of 8.8 months (95% CI: 8.38–9.26), whereas patients with BM and other concurrent metastases had a shorter mOS of 6.0 months (95% CI: 5.75–6.18) (*p* < 0.001) (Table 2, Figure 2).

**Figure 3:**
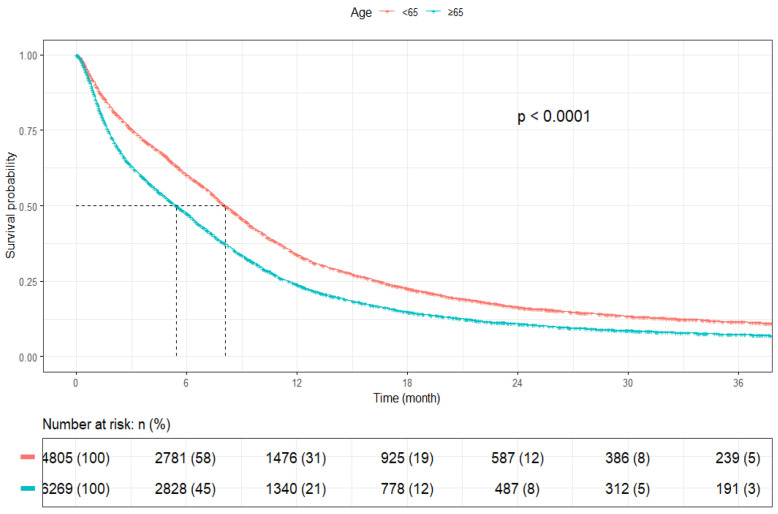
Kaplan–Meier plot by age category.

**Figure 4:**
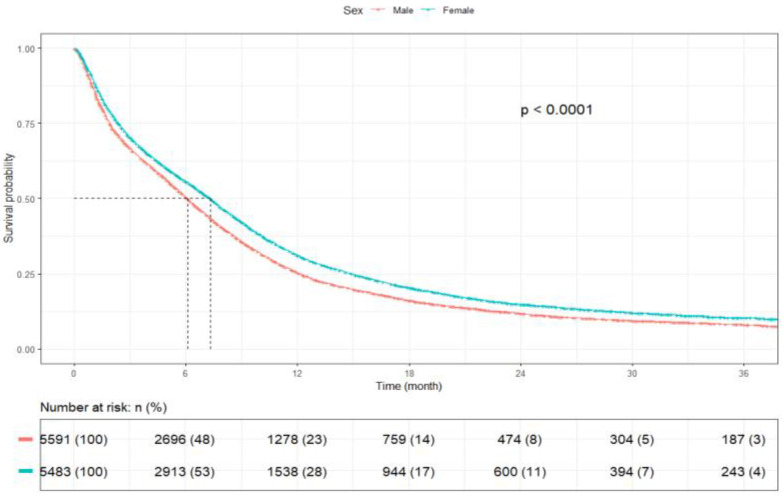
Kaplan–Meier plot by gender.

The mOS for patients aged <65 years was 8.1 months (95% CI: 7.8–8.5), whereas patients aged ≥65 years had shorter mOS of 5.4 months (95% CI: 5.2–5.7) (*p* < 0.001) (Figure 3). Female patients had a greater mOS of 7.3 months (95% CI: 7.0–7.6) as compared to male patients [6.1 months (95% CI: 5.8–6.3)] (*p* < 0.001) (Figure 4).

**Figure 5:**
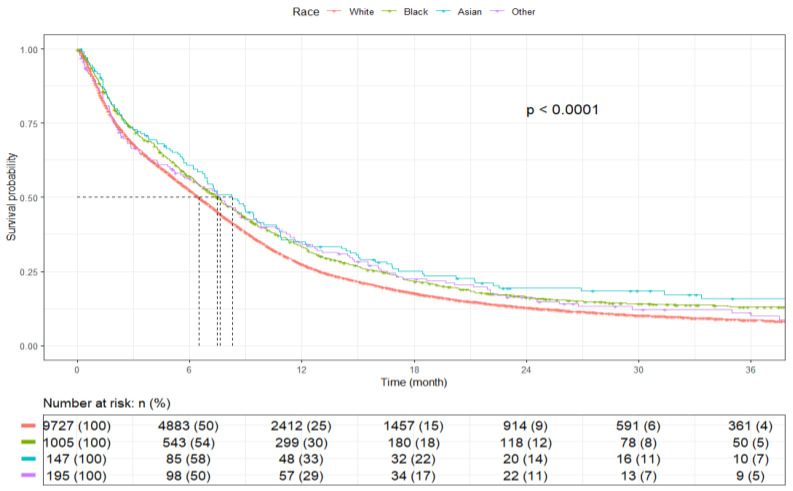
Kaplan–Meier plot by race.

**Figure 6:**
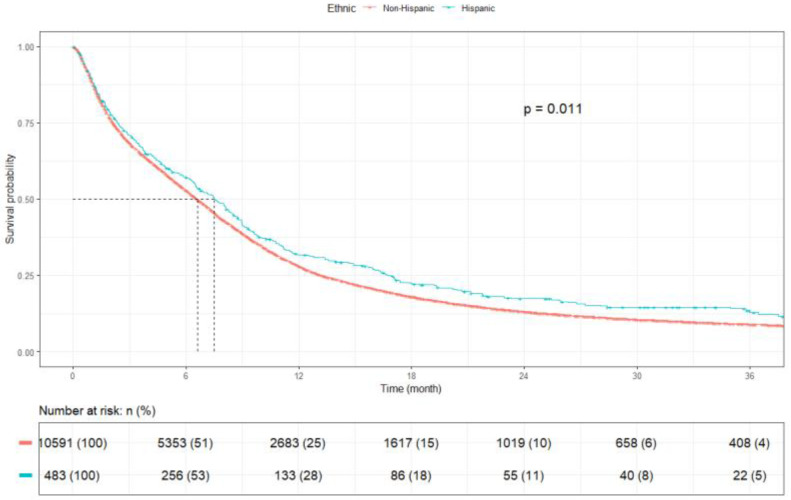
Kaplan Meier plot by ethnicity.

Patients of Asian race had the longest mOS of 8.3 months (95% CI: 6.8–9.9), followed by Black patients at 7.5 months (95% CI: 6.8–8.2), White patients at 6.5 months (95% CI: 6.3–6.7), and other races at 7.6 months (95% CI: 5.7–9.6) (*p* < 0.001) (Figure 5). Patients who identified as non-Hispanic had an mOS of 6.6 months (95% CI: 6.4–6.8), and Hispanics had a slightly greater mOS of 7.5 months (95% CI: 6.5–8.5) (*p* = 0.011) (Figure 6).

**Figure 7:**
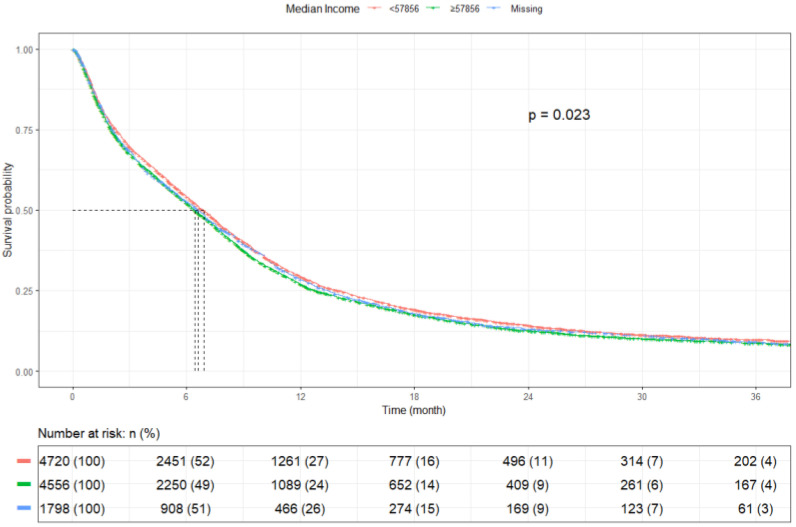
Kaplan Meier plot by median income.

**Figure 8:**
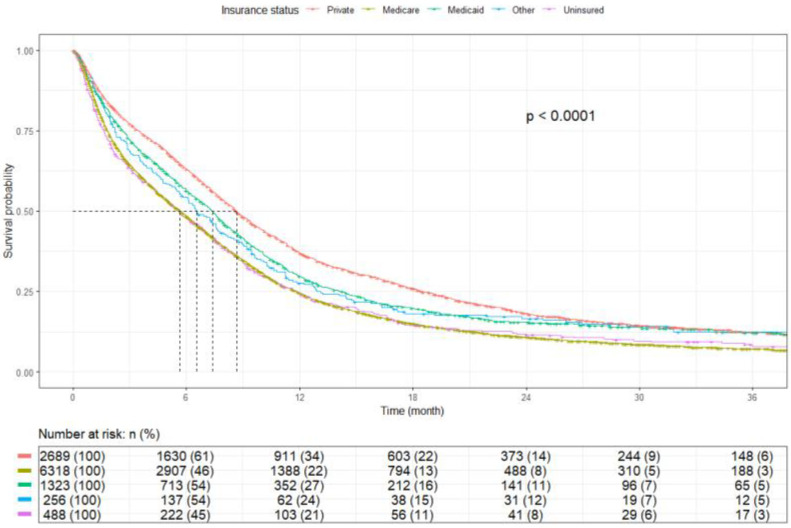
Kaplan–Meier plot by insurance.

**Figure 9:**
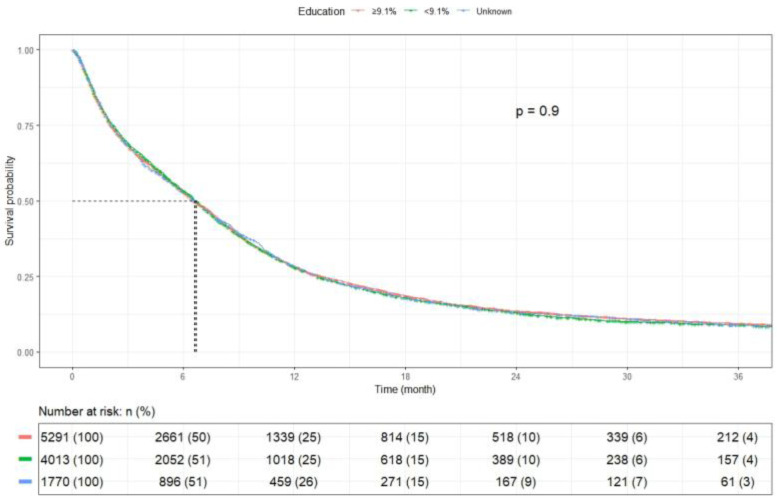
Kaplan–Meier plot by education.

Patients with an annual income ≥$57,856 had an mOS of 6.9 months (95% CI: 6.6–7.2), while those with an annual income <$57,856 had an mOS of 6.4 months (95% CI: 6.2–6.7) (*p* = 0.023) (Figure 7). Private-insured patients had the highest mOS of 8.7 months (95% CI: 8.3–9.1), whereas uninsured patients had worse mOS of 5.6 months (95% CI: 4.7–6.7) (*p* < 0.001). Medicaid-insured and Medicare patients had an mOS of 7.4 months (95% CI: 6.8–7.8) and 5.7 months (95% CI: 5.4–6.0), respectively, while those with other insurances had an mOS of 6.5 months (95% CI: 5.6–7.8) (*p* < 0.0001) (Figure 8). Patients residing in ZIP codes where ≥9.1% of adults aged ≥25 years did not complete high school had an mOS of 6.6 months (95% CI, 6.3–6.9), compared with 6.7 months (95% CI, 6.4–7.0) for those from areas with <9.1% without a high-school diploma (*p* = 0.903) (Figure 9).

**Figure 10:**
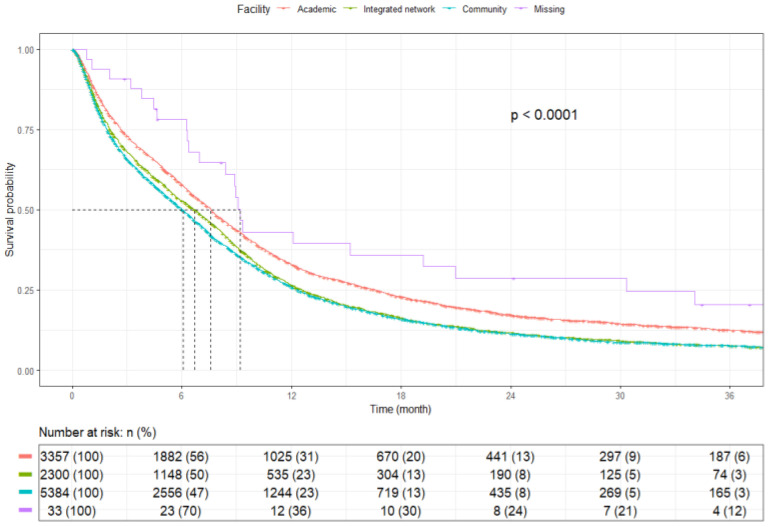
Kaplan–Meier plot by treatment facility type.

Patients treated in an academic facility had a significantly higher mOS of 7.6 months (95% CI: 7.3–7.9) than those treated in an integrated network [6.7 months (95% CI: 6.2–7.1)], while patients treated in a community facility had the lowest mOS of 6.0 months (95% CI: 5.8–6.3) (*p* < 0.0001) (Figure 10).

**Figure 11:**
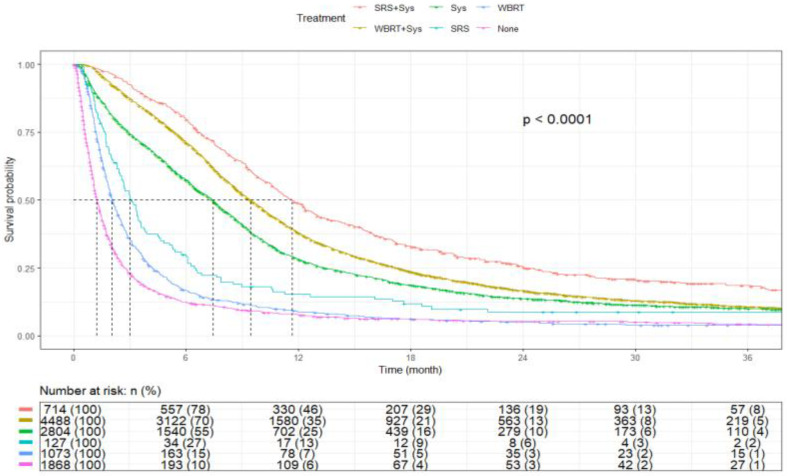
Kaplan–Meier plot by treatment modality.

Across treatment modalities, mOS varied from 2.0 to 11.7 months (*p* < 0.001). Patients who received SRS with Sys had an mOS of 11.7 months (95% CI, 10.9–12.6), followed by WBRT with Sys at 9.4 months (95% CI, 9.1–9.7) and Sys alone at 7.4 months (95% CI, 7.1–7.7). Median survival was 3.0 months (95% CI, 2.6–3.6) for SRS alone, 2.0 months (95% CI, 1.9–2.2) for WBRT alone, and 1.2 months (95% CI, 1.2–1.3) among patients who did not receive active treatment (<0.0001) (Figure 11). Median survival estimates by age, sex, race, ethnicity, insurance, income, and treatment are summarized in Table 3.

### 3.3. Factors Associated with Overall Survival (Multivariable Analysis)

On multivariable analysis, among all patients, age ≥ 65 years was associated with a higher hazard of death compared to younger patients (HR = 1.13; 95% CI, 1.07–1.19; *p* < 0.001). Female sex was associated with lower hazard (HR = 0.87; 95% CI, 0.84–0.91; *p* < 0.001). Compared with White patients, the hazard was lower among Asian (HR = 0.80; 95% CI, 0.67–0.97; *p* = 0.022) and Black patients (HR = 0.88; 95% CI, 0.82–0.95; *p* = 0.001), while no difference was observed for other races (HR = 0.93; 95% CI, 0.79–1.10; *p* = 0.393). Hispanic ethnicity was associated with lower hazard relative to non-Hispanic patients (HR = 0.87; 95% CI, 0.78–0.96; *p* = 0.008).

Higher comorbidity burden corresponded to worse survival (Charlson–Deyo = 1: HR = 1.12; 95% CI, 1.06–1.17; *p* < 0.001; Charlson–Deyo = 2–3: HR = 1.21; 95% CI, 1.14–1.28; *p* < 0.001). Patients with Medicare (HR = 1.12; 95% CI, 1.06–1.20; *p* < 0.001), Medicaid (HR = 1.14; 95% CI, 1.06–1.22; *p* = 0.001), or no insurance (HR = 1.26; 95% CI, 1.13–1.40; *p* < 0.001) had higher hazards than those with private insurance. Lower median household income (<$57,856) was associated with a modestly higher hazard (HR = 1.07; 95% CI, 1.02–1.13; *p* = 0.011). Whereas, educational attainment, defined by the proportion of adults without a high school degree in the patient’s ZIP code ( < 9.1%), was not significantly associated with survival (HR = 1.03; 95% CI, 0.98–1.09; *p* = 0.198).

Concerning facility type, higher hazards were observed for patients treated at integrated networks (HR = 1.15; 95% CI, 1.09–1.22; *p* < 0.001) and community centers (HR = 1.18; 95% CI, 1.12–1.24; *p* < 0.001) compared with academic facilities.

Regarding treatment modalities, hazard ratios for overall survival were as follows: WBRT + Sys = 1.19 (95% CI, 1.09–1.30; *p* < 0.001), Sys alone = 1.44 (95% CI, 1.31–1.58; *p* < 0.001), SRS alone = 2.64 (95% CI, 2.15–3.24; *p* < 0.001), WBRT alone = 3.76 (95% CI, 3.39–4.18; *p* < 0.001), and no treatment = 4.86 (95% CI, 4.40–5.36; *p* < 0.001). Presence of extracranial metastases was also associated with higher hazard (HR = 1.63; 95% CI, 1.55–1.70; *p* < 0.001).

The Cox multivariate analyses findings are presented in Table 4.

### 3.4. Subgroup Analyses by Extent of Metastatic Disease

In Cox multivariate analyses, among patients with SCLC BM only (N = 3611), older age (≥65 years) was associated with reduced survival (HR = 1.28, 95% CI: 1.15–1.42, *p* < 0.001) compared to age <65 years. Compared to White patients, Asians (HR = 0.73, 95% CI: 0.52–1.02, *p* = 0.061) and Blacks (HR = 0.85, 95% CI: 0.75–0.97, *p* = 0.015) had improved survival, although improved survival in Asians was insignificant. Privately insured patients had better survival outcomes compared to those with Medicare (HR = 1.15, 95% CI: 1.02–1.29, *p* = 0.018) and Medicaid (HR = 1.29, 95% CI: 1.12–1.47, *p* < 0.001). All treatment modalities, including WBRT+Sys (HR = 1.19, 95% CI: 1.03–1.38, *p* = 0.022), Sys (HR = 1.29, 95% CI: 1.10–1.52, *p* = 0.002), SRS (HR = 2.61, 95% CI: 1.97–3.47, *p* < 0.001), and WBRT (HR = 3.55, 95% CI: 3.00–4.21, *p* < 0.001), showed worse outcomes compared to SRS+Sys. The presence of comorbidities yielded worse survival outcomes. Hazard ratios estimated from the Cox multivariate analyses for sex, ethnicity, income, education, and distance to treatment facility were insignificant (Table 5).

For SCLC BM patients with other concurrent metastases (N = 7463), patients aged ≥65 years also had reduced survival (HR = 1.07, 95% CI: 1.00–1.15, *p* = 0.039). Patients treated with SRS+Sys showed better survival outcomes compared to other treatment modalities, including WBRT+Sys (HR = 1.19, 95% CI: 1.07–1.34, *p* = 0.002), Sys (HR = 1.51, 95% CI: 1.34–1.70, *p* < 0.001), SRS (HR = 2.61, 95% CI: 1.91–3.55, *p* < 0.001), and WBRT alone (HR = 3.91, 95% CI: 3.41–4.47, *p* < 0.001). Female sex (HR = 0.84, 95% CI: 0.80–0.89; *p* < 0.001) and Hispanic ethnicity (HR = 0.80, 95% CI: 0.70–0.92; *p* = 0.001) were associated with improved survival. Hazard ratios estimated from the cox multivariate analyses for race, education, and distance to the treatment facility were insignificant (Table 6).

### 3.5. Assessment of Proportional Hazards and AFT Model Validation

On visual inspection of scaled Schoenfeld residuals, we noted that the PH assumption seemed to have been generally satisfied across covariates, with residuals fluctuating randomly around zero without systematic deviation. Subtle time-dependent trends were observed for sex (gradual upward drift) and metastatic extent (minor divergence at later follow-up), indicating limited non-proportionality (Supplementary Figure S1).

To evaluate the robustness of the survival estimates, an AFT model was fitted as a complementary approach (Supplementary Table S1). The AFT model yielded directionally consistent results with the Cox model. Shorter survival times were associated with older age (≥65 years; TR = 0.88, *p* < 0.001), higher comorbidity burden (TR = 0.82, *p* < 0.001), Medicare or Medicaid insurance (TR = 0.86, *p* < 0.001), lower income (TR = 0.92, *p* = 0.004), non-academic treatment facilities (TR = 0.82/0.84, *p* < 0.001), and the presence of extracranial metastases (TR = 0.58, *p* < 0.001). Conversely, female sex (TR = 1.16, *p* < 0.001), Black (TR = 1.14, *p* = 0.001) and Asian race (TR = 1.26, *p* = 0.020), and Hispanic ethnicity (TR = 1.18, *p* = 0.004) were associated with prolonged survival. Treatment modality remained a strong determinant of clinical outcome, with the shortest survival observed among patients receiving WBRT alone (TR = 0.25) or no treatment (TR = 0.20).

Overall, these AFT findings corroborated the Cox model results, indicating that minor deviations from proportionality in select covariates did not materially influence the direction or significance of effects.

## 4. Discussion

Despite advances in lung cancer management over the past decade, meaningful variability in outcomes persists. Using data from the National Cancer Database (NCDB, 2018–2020), this study evaluated contemporary factors associated with survival among patients with SCLC and BM. Age, sex, ethnicity, insurance status, household income, treatment facility type, distance to care, and treatment modality were independently associated with OS on multivariable analysis. To our knowledge, this represents one of the largest contemporary evaluations of clinical and treatment-related factors influencing survival in this patient population.

This study found that patients <65 years with SCLC BM have better survival outcomes than older patients (≥65 years). These findings align with the results from a study conducted by Joanna et al., who concluded that treatment response and survival rates were lower with advancing age in limited-stage SCLC [25]. Similarly, Wang et al. found that older patients diagnosed with stage 3 SCLC had worse OS [26], possibly attributed to suboptimal treatment, comorbidities, and poor performance status, as older patients were less likely to be treated with chemoradiotherapy, intensive chemotherapy, and prophylactic cranial irradiation [25]. Wang et al. used subgroup analyses to conclude that younger patients tended to have better survival outcomes independent of their radiation therapy status [26]. Consistent with other studies on limited and extensive stage SCLC, which used NCDB and the Surveillance, Epidemiology, and End Results (SEER) databases, a survival advantage was observed for females compared to males [18,27,28]. Wang et al. suggest that this difference may be attributed to intrinsic genomic factors [28]. These results show that age and sex independently influence survival in patients with SCLC and BM.

The current study found that patients of Asian and Black race with SCLC and BM demonstrated longer survival compared with White patients. Zhou et al. and Roof et al., using NCDB data, similarly reported improved survival among Asian and Black patients in limited- and extensive-stage SCLC, respectively [18,27]. In contrast, Albain et al., analyzing data from the Southwest Oncology Group (SWOG), observed better survival outcomes in White patients; however, these differences were attenuated when more recent clinical trials were included [29]. Studies by Sai-Hong Ignatius et al. in extensive-disease SCLC and Uprety et al. in metastatic NSCLC also described better outcomes among White patients [19,30], whereas Biswas et al. found that race was not independently associated with survival in stage I NSCLC [16].

Haddad et al. noted that survival differences across racial groups in lung cancer diminish after adjusting for stage at diagnosis and treatment access [31]. Similarly, Blackstock et al. reported comparable outcomes between Black and non-Black patients with extensive-stage SCLC when treated with equivalent regimens [32]. These observations suggest that discrepancies reported across studies may reflect differences in patient selection, study design, and adjustment for clinical and treatment variables rather than inherent racial effects [33,34,35]. Biological heterogeneity may also contribute. Population-level differences in drug-metabolizing gene polymorphisms could influence systemic therapy response; for instance, irinotecan plus cisplatin improved OS in a Japanese randomized clinical trial [36] but not in a comparable U.S. trial [37]. Differences in how prior studies adjusted for confounding factors such as stage at diagnosis and clinical covariates may also contribute to inconsistent findings across datasets. Genetic variation and disease biology among lung cancer subtypes could further explain these differences.

In the present analysis, Black females demonstrated the longest mOS among all subgroups, suggesting potential biological or treatment-related influences that warrant further evaluation. This study also identified improved survival among Hispanic patients compared with non-Hispanic patients. Similar trends were observed by Ou et al., who reported superior one- and two-year survival in Hispanic patients with extensive-stage SCLC [19]. Klugman et al. further showed that, after adjusting for clinical factors and smoking status, both Hispanics and Asians had improved survival compared with non-Hispanic Whites in lung cancer overall [38]. Zhou et al. reported comparable radiation utilization across groups but found higher chemotherapy receipt among Hispanic patients, which may have contributed to longer survival [18]. Conversely, Tapan et al. observed that improved survival among Hispanics with extensive-stage SCLC was not directly attributable to chemotherapy exposure [39]. Although genetic and pharmacogenomic factors may underlie some of these patterns, cultural and behavioral influences, such as family involvement, treatment adherence, and decision-making preferences, could also play a role. Further investigation integrating genomic, treatment, and patient-reported data is needed to better delineate the mechanisms contributing to these observed differences in outcomes.

Patients with private insurance had better survival outcomes than those with Medicare, Medicaid, and uninsured patients in the current study. This finding is similar to studies on limited and extensive-stage SCLC [18,40]. A retrospective study using the NCDB that included both limited and extensive stage SCLC reported that Medicaid coverage was not associated with a survival advantage compared with being uninsured. This may be due to treatment delays, limited access to specialists, and lower rates of standard care, highlighting the need for policy improvements. This is also true for NSCLC [41,42]. It has been shown that, even when presenting with the same cancer stage, publicly insured patients are significantly less likely than privately insured patients to receive guideline-concordant treatment such as systemic therapy in advanced-stage NSCLC and surgical resection in early-stage NSCLC [42,43].

In the present study, patients with private insurance had longer OS compared with those covered by Medicare or Medicaid, as well as uninsured patients. Similar trends have been reported in studies of both limited- and extensive-stage SCLC [18,40]. A retrospective NCDB analysis encompassing all SCLC stages found that Medicaid coverage did not confer a survival advantage compared with being uninsured, a pattern attributed to treatment delays, limited access to oncology specialists, and lower rates of standard-of-care therapy. Comparable findings have been described in NSCLC, where publicly insured patients are significantly less likely than privately insured patients to receive guideline-concordant treatments, including systemic therapy in advanced-stage disease and surgical resection in early-stage settings [41,42,43].

Survival differences were also observed according to treatment facility type, with patients treated at academic centers demonstrating better outcomes than those managed in community or integrated network facilities. In addition, patients residing in higher-income areas had improved survival. These findings are consistent with prior studies in SCLC [18,27] and NSCLC [44], although the present analysis specifically addresses SCLC with brain metastases. The association between income and survival may reflect broader access to comprehensive care, specialized expertise, and timely initiation of multimodality treatment, all of which are known to influence outcomes in cancer care.

The current study also found improved survival among patients receiving SRS + Sys compared with other treatment modalities. WBRT has traditionally been the standard approach for BM; however, SRS has increasingly replaced WBRT in appropriately selected patients with limited intracranial disease [45]. An NCDB analysis similarly reported superior outcomes with upfront SRS relative to WBRT, although this finding may in part reflect selection bias favoring patients with better performance status or lower intracranial disease burden [46]. Utilization of SRS has also been linked to institutional and temporal factors, including treatment facility type, educational level, and study period, suggesting evolving practice patterns and broader adoption in contemporary management [47].

### Limitations and Future Directions

This study has certain limitations. The NCDB captures first-course treatment only, defined as therapies delivered before disease progression or recurrence; therefore, later-line systemic therapy, salvage radiation, repeat SRS, or sequential use of SRS and WBRT cannot be evaluated. Because of this structure, treatment categories appear mutually exclusive, which may not fully reflect real-world practice, where modalities can be used at different time points. In addition, the database does not include longitudinal treatment details, radiation dose or fractionation, specific systemic therapy regimens, biomarker information, measures of intracranial disease burden, or cause of death.

Despite these constraints, the large national cohort provides meaningful insight into patterns of care and survival in SCLC with BM. Future studies should incorporate molecular and genetic biomarkers to better characterize biological drivers of survival, use longitudinal or follow-up designs to capture treatment sequencing and temporal trends, and continue expanding sample sizes to strengthen statistical power. The findings from this study may also serve as a foundation for evaluating emerging and comparative treatment strategies in this population.

## 5. Conclusions

In this contemporary, national cohort of patients with SCLC and brain metastases (2018–2020 NCDB), we describe current treatment patterns and survival. Younger age and female sex were associated with longer overall survival, and combined stereotactic radiosurgery with systemic therapy (SRS + Sys) was associated with notable adjusted survival relative to other initial strategies. Outcomes were also more favorable for patients treated at academic centers. Signals by race/ethnicity were observed but remain variable across datasets and are likely multifactorial.

These findings are observational and hypothesis-generating. They support careful patient selection for focal radiotherapy in combination with systemic therapy, and they highlight the need for prospective studies that incorporate performance status, intracranial disease burden, radiotherapy parameters, and specific systemic regimens to define optimal sequencing and benefit. The data provide contemporary benchmarks to inform trial design and clinical counseling in SCLC with brain metastases.

## Figures and Tables

**Table 1:** Patient demographics and baseline characteristics (N = 11,074).

Characteristics	Level	All (N = 11,074)	SCLC with BM Only (N = 3611)	SCLC-BM with Other Concurrent Metastases (N = 7463)
Age	Median (IQR)	66.0 (60.0 to 73.0)	66.0 (60.0 to 73.0)	66.0 (60.0 to 72.0)
	<65 years	4805 (43.4)	1513 (41.9)	3292 (44.1)
	≥65 years	6269 (56.6)	2098 (58.1)	4171 (55.9)
Sex	Male	5591 (50.5)	1697 (47.0)	3894 (52.2)
	Female	5483 (49.5)	1914 (53.0)	3569 (47.8)
Race	White	9727 (87.8)	3089 (85.5)	6638 (88.9)
	Black	1005 (9.1)	398 (11.0)	607 (8.1)
	Asian	147 (1.3)	54 (1.5)	93 (1.2)
	Other	195 (1.8)	70 (1.9)	125 (1.7)
Ethnicity	Non-Hispanic	10,591 (95.6)	3441 (95.3)	7150 (95.8)
	Hispanic	483 (4.4)	170 (4.7)	313 (4.2)
Charlson–Deyo Comorbidity Index	0	6354 (57.4)	2064 (57.2)	4290 (57.5)
	1	2659 (24.0)	868 (24.0)	1791 (24.0)
	2–3	2061 (18.6)	679 (18.8)	1382 (18.5)
Insurance	Private	2689 (24.3)	830 (23.0)	1859 (24.9)
	Medicare	6318 (57.1)	2085 (57.7)	4233 (56.7)
	Medicaid	1323 (11.9)	450 (12.5)	873 (11.7)
	Other	256 (2.3)	80 (2.2)	176 (2.4)
	Uninsured	488 (4.4)	166 (4.6)	322 (4.3)
Median Income *	≥$57,856	4720 (42.6)	1512 (41.9)	3208 (43.0)
	<$57,856	4556 (41.1)	1528 (42.3)	3028 (40.6)
	Unknown	1798 (16.2)	571 (15.8)	1227 (16.4)
Education **	≥9.1%	5291 (47.8)	1806 (50.0)	3485 (46.7)
	<9.1%	4013 (36.2)	1245 (34.5)	2768 (37.1)
	Unknown	1770 (16.0)	560 (15.5)	1210 (16.2)
Facility type	Academic	3357 (30.3)	1145 (31.7)	2212 (29.6)
	Integrated network	2300 (20.8)	732 (20.3)	1568 (21.0)
	Community	5384 (48.6)	1725 (47.8)	3659 (49.0)
	Unknown	33 (0.3)	9 (0.2)	24 (0.3)
Distance (Crowfly) ***	11.2+ miles	4713 (42.6)	1585 (43.9)	3128 (41.9)
	<11.2 miles	4666 (42.1)	1486 (41.2)	3180 (42.6)
	Missing	1695 (15.3)	540 (15.0)	1155 (15.5)
Year of diagnosis	2018	3825 (34.5)	1270 (35.2)	2555 (34.2)
	2019	3827 (34.6)	1242 (34.4)	2585 (34.6)
	2020	3422 (30.9)	1099 (30.4)	2323 (31.1)
Treatment	SRS+Sys	714 (6.4)	298 (8.3)	416 (5.6)
	WBRT+Sys	4488 (40.5)	1469 (40.7)	3019 (40.5)
	Sys	2804 (25.3)	696 (19.3)	2108 (28.2)
	SRS	127 (1.1)	76 (2.1)	51 (0.7)
	WBRT	1073 (9.7)	453 (12.5)	620 (8.3)
	None	1868 (16.9)	619 (17.1)	1249 (16.7)
Vital status	Alive	1678 (15.2)	777 (21.5)	901 (12.1)
	Dead	9396 (84.8)	2834 (78.5)	6562 (87.9)
Median follow-up	Median (IQR)	34.2 (24.6 to 44.4)	34.7 (25.1 to 44.6)	33.7 (24.3 to 44.3)

Abbreviations: BM, brain metastasis; SCLC, small cell lung cancer; SRS, stereotactic radiosurgery; Sys, systemic therapy; WBRT, whole-brain radiotherapy. * Median Income: Area-level median household income linked to patient ZIP codes using American Community Survey 2016–2020 data; analyzed as ≥$57,856 vs. <$57,856. ** Education: Percentage of adults aged ≥25 years within the patient’s ZIP code who did not complete high school; categorized as ≥9.1% vs. <9.1%. *** Distance (Crowfly): Straight-line (“crow-fly”) distance in miles between the centroid of the patient’s residential ZIP code and the treating facility; analyzed as <11.2 vs. ≥11.2 miles.

**Table 2:** Median OS and survival rates for patients with SCLC with BM.

Cohort	Median (Months)	3-month Survival Rate (%)	6-month Survival Rate (%)	1-year Survival Rate (%)	2-year Survival Rate (%)	3-year Survival Rate (%)	*p*
All patients (N = 11,074)	6.60 (6.47–6.87)	68.6 (67.7–69.4)	53.1 (52.2–54.1)	28.2 (27.4–29.1)	13.4 (12.7–14.1)	9.2 (8.6–9.8)	<0.0001
SCLC with BM only (N = 3611)	8.80 (8.38–9.26)	73.6 (72.2–75.1)	60.0 (58.4–61.6)	39.0 (37.4–40.7)	21.3 (19.9–22.7)	15.4 (14.1–16.9)	
SCLC BM with other Concurrent metastases (N = 7463)	5.95 (5.75–6.18)	66.1 (65.1–67.2)	49.8 (48.7–51.0)	23.0 (22.0–24.0)	9.5 (8.8–10.2)	6.1 (5.5–6.8)	

Abbreviations: SCLC: Small cell lung cancer; BM: Brain Metastases.

**Table 3:** Median OS and survival rates by age, gender, race, ethnicity, insurance status, income, education, and treatment.

Cohort	Level	Number	Median OS (95%CI)	*p*-Value
All patients	All patients	11,074	6.6 (6.5–6.9)	-
Age	<65 years	4805	8.1 (7.8–8.5)	<0.0001
	≥65 years	6269	5.4 (5.2–5.7)	
Gender	Male	5591	6.1 (5.8–6.3)	<0.0001
	Female	5483	7.3 (7.0–7.6)	
Race	White	9727	6.5 (6.3–6.7)	<0.0001
	Black	1005	7.5 (6.8–8.2)	
	Asian	147	8.3 (6.8–9.9)	
	Other	195	7.6 (5.7–9.6)	
Ethnicity	Non-Hispanic	10,591	6.6 (6.4–6.8)	0.011
	Hispanic	483	7.5 (6.5–8.5)	
Charlson–Deyo Comorbidity Index	0	6354	7.4 (7.2–7.7)	<0.0001
	1	2659	6.3 (6.0–6.6)	
	2–3	2061	4.9 (4.5–5.3)	
Median Income *	≥$57,856	4720	6.9 (6.6–7.2)	0.023
	<$57,856	4556	6.4 (6.2–6.7)	
	Unknown	1798	6.6 (6.1–7.0)	
Education **	≥9.1%	5291	6.6 (6.3–6.9)	0.903
	<9.1%	4013	6.7 (6.4–7.0)	
	Unknown	1770	6.6 (6.1–7.1)	
Insurance	Private	2689	8.7 (8.3–9.1)	<0.0001
	Medicare	6318	5.7 (5.4–6.0)	
	Medicaid	1323	7.4 (6.8–7.8)	
	Other	256	6.5 (5.6–7.8)	
	Uninsured	488	5.6 (4.7–6.7)	
Facility	Academic	3357	7.6 (7.3–7.9)	<0.0001
	Integrated network	2300	6.7 (6.2–7.1)	
	Community	5384	6.0 (5.8–6.3)	
	Unknown	33	9.2 (6.9–30.3)	
Crowfly distance ***	<11.2 miles	4666	6.4 (6.2–6.7)	0.601
	11.2+ miles	4713	6.9 (6.6–7.2)	
	Missing	1695	6.6 (6.1–7.1)	
Treatment	SRS+Sys	714	11.7 (10.9–12.6)	<0.0001
	WBRT+Sys	4488	9.4 (9.1–9.7)	
	Sys	2804	7.4 (7.1–7.7)	
	SRS	127	3.0 (2.6–3.6)	
	WBRT	1073	2.0 (1.9–2.2)	
	None	1868	1.2 (1.2–1.3)	

Abbreviations: OS, overall survival; SRS, stereotactic radiosurgery; Sys, systemic therapy; WBRT, whole-brain radiotherapy. * Median Income: Area-level median household income linked to patient ZIP codes using American Community Survey 2016–2020 data; analyzed as ≥$57,856 vs. <$57,856. ** Education: Percentage of adults aged ≥25 years within the patient’s ZIP code who did not complete high school; categorized as ≥9.1% vs. <9.1%. *** Distance (Crowfly): Straight-line (“crow-fly”) distance in miles between the centroid of the patient’s residential ZIP code and the treating facility; analyzed as <11.2 vs. ≥11.2 miles.

**Table 4:** Multivariable Cox proportional hazards analysis of overall survival among all patients with small cell lung cancer and brain metastases (N = 11,074).

Characteristics	Level	N (%)	HR (Univariable)	HR (Multivariable)
Age	<65 Years	4805 (43.4)	-	-
	≥65 Years	6269 (56.6)	1.33 (1.28–1.39, *p* < 0.001)	1.13 (1.07–1.19, *p* < 0.001)
Sex	Male	5591 (50.5)	-	-
	Female	5483 (49.5)	0.87 (0.83–0.90, *p* < 0.001)	0.87 (0.84–0.91, *p* < 0.001)
Race	White	9727 (87.8)	-	-
	Black	1005 (9.1)	0.86 (0.80–0.92, *p* < 0.001)	0.88 (0.82–0.95, *p* = 0.001)
	Asian	147 (1.3)	0.77 (0.65–0.93, *p* = 0.006)	0.80 (0.67–0.97, *p* = 0.022)
	Other	195 (1.8)	0.90 (0.77–1.06, *p* = 0.214)	0.93 (0.79–1.10, *p* = 0.393)
Ethnicity	Non-Hispanic	10,591 (95.6)	-	-
	Hispanic	483 (4.4)	0.88 (0.79–0.97, *p* = 0.011)	0.87 (0.78–0.96, *p* = 0.008)
Charlson–Deyo Comorbidity Index	0	6354 (57.4)	-	-
	1	2659 (24.0)	1.11 (1.05–1.16, *p* < 0.001)	1.12 (1.06–1.17, *p* < 0.001)
	2–3	2061 (18.6)	1.30 (1.24–1.38, *p* < 0.001)	1.21 (1.14–1.28, *p* < 0.001)
Insurance	Private	2689 (24.3)	-	-
	Medicare	6318 (57.1)	1.40 (1.33–1.47, *p* < 0.001)	1.12 (1.06–1.20, *p* < 0.001)
	Medicaid	1323 (11.9)	1.14 (1.06–1.23, *p* < 0.001)	1.14 (1.06–1.22, *p* = 0.001)
	Other	256 (2.3)	1.18 (1.03–1.36, *p* = 0.018)	0.89 (0.77–1.03, *p* = 0.119)
	Uninsured	488 (4.4)	1.40 (1.26–1.56, *p* < 0.001)	1.26 (1.13–1.40, *p* < 0.001)
Median Income *	≥$57,856	4720 (42.6)	-	-
	<$57,856	4556 (41.1)	1.06 (1.02–1.11, *p* = 0.006)	1.07 (1.02–1.13, *p* = 0.011)
	Unknown	1798 (16.2)	1.03 (0.97–1.10, *p* = 0.271)	1.34 (0.90–2.00, *p* = 0.155)
Education **	≥9.1%	5291 (47.8)	-	-
	<9.1%	4013 (36.2)	1.01 (0.97–1.06, *p* = 0.651)	1.03 (0.98–1.09, *p* = 0.198)
	Unknown	1770 (16.0)	1.01 (0.95–1.07, *p* = 0.856)	0.67 (0.42–1.08, *p* = 0.101)
Facility Type	Academic	3357 (30.3)	-	-
	Integrated network	2300 (20.8)	1.20 (1.13–1.27, *p* < 0.001)	1.15 (1.09–1.22, *p* < 0.001)
	Community	5384 (48.6)	1.25 (1.19–1.31, *p* < 0.001)	1.18 (1.12–1.24, *p* < 0.001)
	Unknown	33 (0.3)	0.72 (0.48–1.07, *p* = 0.103)	0.77 (0.51–1.15, *p* = 0.200)
Distance ***	11.2+ miles	4713 (42.6)	0.98 (0.94–1.02, *p* = 0.330)	1.00 (0.96–1.04, *p* = 0.965)
	<11.2 miles	4666 (42.1)	-	-
	Missing	1695 (15.3)	1.00 (0.94–1.06, *p* = 0.908)	1.13 (0.88–1.46, *p* = 0.344)
Treatment	SRS+Sys	714 (6.4)	-	-
	WBRT+Sys	4488 (40.5)	1.28 (1.17–1.40, *p* < 0.001)	1.19 (1.09–1.30, *p* < 0.001)
	Sys	2804 (25.3)	1.59 (1.45–1.74, *p* < 0.001)	1.44 (1.31–1.58, *p* < 0.001)
	SRS	127 (1.1)	2.58 (2.10–3.17, *p* < 0.001)	2.64 (2.15–3.24, *p* < 0.001)
	WBRT	1073 (9.7)	3.82 (3.43–4.24, *p* < 0.001)	3.76 (3.39–4.18, *p* < 0.001)
	None	1868 (16.9)	5.17 (4.69–5.70, *p* < 0.001)	4.86 (4.40–5.36, *p* < 0.001)
Other Concurrent Metastases	No	3611 (32.6)	-	-
	Yes	7463 (67.4)	1.50 (1.44–1.57, *p* < 0.001)	1.63 (1.55–1.70, *p* < 0.001)

Abbreviations: HR, hazard ratio; WBRT, whole-brain radiation therapy; SRS, stereotactic radiosurgery; Sys, systemic therapy; CI, confidence interval. * Median Income: Area-level median household income linked to patient ZIP codes using American Community Survey 2016–2020 data; analyzed as ≥$57,856 vs. <$57,856. ** Education: Percentage of adults aged ≥25 years within the patient’s ZIP code who did not complete high school; categorized as ≥9.1% vs. <9.1%. *** Distance (Crowfly): Straight-line (“crow-fly”) distance in miles between the centroid of the patient’s residential ZIP code and the treating facility; analyzed as <11.2 vs. ≥11.2 miles.

**Table 5:** Multivariable Cox proportional hazards analysis of overall survival among patients with small cell lung cancer and brain-only metastases (BM-only cohort) (N = 3611).

Characteristics	Level	N (%)	HR (Univariable)	HR (Multivariable)
Age	<65 Years	1513 (41.9)	-	-
	≥65 Years	2098 (58.1)	1.52 (1.41–1.63, *p* < 0.001)	1.28 (1.15–1.42, *p *< 0.001)
Sex	Male	1697 (47.0)	-	-
	Female	1914 (53.0)	0.95 (0.88–1.02, *p* = 0.139)	0.94 (0.87–1.02, *p* = 0.119)
Race	White	3089 (85.5)	-	-
	Black	398 (11.0)	0.82 (0.73–0.93, *p* = 0.002)	0.85 (0.75–0.97, *p *= 0.015)
	Asian	54 (1.5)	0.66 (0.48–0.92, *p* = 0.013)	0.73 (0.52–1.02, *p* = 0.061)
	Other	70 (1.9)	0.92 (0.70–1.20, *p* = 0.546)	0.91 (0.69–1.21, *p* = 0.527)
Ethnicity	Non-Hispanic	3441 (95.3)	-	-
	Hispanic	170 (4.7)	0.97 (0.82–1.16, *p* = 0.757)	0.99 (0.82–1.19, *p* = 0.898)
Comorbid Condition	0	2064 (57.2)	-	-
	1	868 (24.0)	1.08 (0.99–1.18, *p* = 0.098)	1.11 (1.02–1.22, *p* = 0.020)
	2–3	679 (18.8)	1.24 (1.13–1.37, *p* < 0.001)	1.17 (1.06–1.29, *p* = 0.002)
Insurance	Private	830 (23.0)	-	-
	Medicare	2085 (57.7)	1.58 (1.44–1.74, *p* < 0.001)	1.15 (1.02–1.29, *p *= 0.018)
	Medicaid	450 (12.5)	1.23 (1.07–1.40, *p* = 0.003)	1.29 (1.12–1.47, *p* < 0.001)
	Other	80 (2.2)	1.21 (0.93–1.57, *p* = 0.163)	0.85 (0.65–1.11, *p* = 0.226)
	Uninsured	166 (4.6)	1.63 (1.35–1.97, *p* < 0.001)	1.49 (1.23–1.80, *p* < 0.001)
Median Income *	≥$57,856	1512 (41.9)	-	-
	<$57,856	1528 (42.3)	1.08 (1.00–1.17, *p* = 0.053)	1.06 (0.97–1.17, *p* = 0.199)
	Unknown	571 (15.8)	1.13 (1.01–1.25, *p* = 0.031)	1.39 (0.69–2.79, *p* = 0.359)
Education **	≥9.1%	1806 (50.0)	-	-
	<9.1%	1245 (34.5)	1.01 (0.93–1.10, *p* = 0.790)	1.05 (0.96–1.16, *p* = 0.300)
	Unknown	560 (15.5)	1.09 (0.98–1.21, *p* = 0.127)	0.89 (0.38–2.09, *p* = 0.786)
Facility Type	Academic	1145 (31.7)	-	-
	Integrated network	732 (20.3)	1.28 (1.15–1.42, *p* < 0.001)	1.20 (1.08–1.34, *p* = 0.001)
	Community	1725 (47.8)	1.32 (1.21–1.44, *p* < 0.001)	1.23 (1.13–1.35, *p* < 0.001)
	Unknown	9 (0.2)	0.46 (0.17–1.23, *p* = 0.124)	0.38 (0.14–1.03, *p* = 0.057)
Distance (Crowfly) ***	11.2+ miles	1585 (43.9)	0.99 (0.92–1.08, *p* = 0.875)	1.01 (0.93–1.09, *p* = 0.868)
	<11.2 miles	1486 (41.2)	-	-
	Missing	540 (15.0)	1.08 (0.97–1.20, *p* = 0.180)	0.89 (0.54–1.48, *p* = 0.664)
Treatment	SRS+Sys	298 (8.3)	-	-
	WBRT+Sys	1469 (40.7)	1.25 (1.08–1.45, *p* = 0.003)	1.19 (1.03–1.38, *p* = 0.022)
	Sys	696 (19.3)	1.38 (1.18–1.62, *p* < 0.001)	1.29 (1.10–1.52, *p* = 0.002)
	SRS	76 (2.1)	2.97 (2.24–3.93, *p* < 0.001)	2.61 (1.97–3.47, *p *< 0.001)
	WBRT	453 (12.5)	3.81 (3.22–4.50, *p* < 0.001)	3.55 (3.00–4.21, *p *< 0.001)
	None	619 (17.1)	4.66 (3.97–5.48, *p* < 0.001)	4.26 (3.62–5.02, *p *< 0.001)

Abbreviations: HR, hazard ratio; WBRT, whole-brain radiation therapy; SRS, stereotactic radiosurgery; Sys, systemic therapy; CI, confidence interval. * Median Income: Area-level median household income linked to patient ZIP codes using American Community Survey 2016–2020 data; analyzed as ≥$57,856 vs. <$57,856. ** Education: Percentage of adults aged ≥25 years within the patient’s ZIP code who did not complete high school; categorized as ≥9.1% vs. <9.1%. *** Distance (Crowfly): Straight-line (“crow-fly”) distance in miles between the centroid of the patient’s residential ZIP code and the treating facility; analyzed as <11.2 vs. ≥11.2 miles.

**Table 6:** Multivariable Cox proportional hazards analysis of overall survival among patients with small cell lung cancer and concurrent extracranial metastases (other concurrent metastases-only cohort) (N = 7463).

Characteristics	Level	N (%)	HR (Univariable)	HR (Multivariable)
Age	<65 Years	3292 (44.1)	-	-
	≥65 Years	4171 (55.9)	1.27 (1.21–1.33, *p* < 0.001)	1.07 (1.00–1.15, *p *= 0.039)
Sex	Male	3894 (52.2)	-	-
	Female	3569 (47.8)	0.84 (0.80–0.89, *p* < 0.001)	0.84 (0.80–0.89, *p *< 0.001)
Race	White	6638 (88.9)	-	-
	Black	607 (8.1)	0.93 (0.85–1.02, *p* = 0.134)	0.89 (0.82–0.98, *p *= 0.019)
	Asian	93 (1.2)	0.89 (0.71–1.11, *p* = 0.310)	0.87 (0.70–1.09, *p* = 0.236)
	Other	125 (1.7)	0.93 (0.76–1.13, *p* = 0.452)	0.94 (0.76–1.15, *p* = 0.539)
Ethnicity	Non-Hispanic	7150 (95.8)	-	-
	Hispanic	313 (4.2)	0.83 (0.74–0.95, *p* = 0.005)	0.80 (0.70–0.92, *p *= 0.001)
Comorbid Condition	0	4290 (57.5)	-	-
	1	1791 (24.0)	1.13 (1.06–1.20, *p* < 0.001)	1.12 (1.05–1.19, *p* < 0.001)
	2–3	1382 (18.5)	1.36 (1.27–1.45, *p* < 0.001)	1.22 (1.14–1.30, *p *< 0.001)
Insurance	Private	1859 (24.9)	-	-
	Medicare	4233 (56.7)	1.33 (1.25–1.41, *p* < 0.001)	1.11 (1.03–1.20, *p* = 0.005)
	Medicaid	873 (11.7)	1.12 (1.03–1.23, *p* = 0.008)	1.07 (0.98–1.17, *p* = 0.110)
	Other	176 (2.4)	1.18 (1.00–1.39, *p* = 0.052)	0.91 (0.77–1.08, *p* = 0.267)
	Uninsured	322 (4.3)	1.33 (1.17–1.51, *p* < 0.001)	1.16 (1.02–1.32, *p* = 0.026)
Median Income *	≥$57,856	3208 (43.0)	-	-
	<$57,856	3028 (40.6)	1.06 (1.01–1.12, *p* = 0.025)	1.07 (1.01–1.14, *p* = 0.028)
	Unknown	1227 (16.4)	0.99 (0.92–1.06, *p* = 0.707)	1.35 (0.82–2.21, *p* = 0.236)
Education **	≥9.1%	3485 (46.7)	-	-
	<9.1%	2768 (37.1)	0.99 (0.94–1.04, *p* = 0.733)	1.02 (0.96–1.09, *p* = 0.481)
	Unknown	1210 (16.2)	0.95 (0.89–1.02, *p* = 0.164)	0.62 (0.35–1.09, *p* = 0.096)
Facility Type	Academic	2212 (29.6)	-	-
	Integrated network	1568 (21.0)	1.15 (1.07–1.23, *p* < 0.001)	1.12 (1.05–1.21, *p* = 0.001)
	Community	3659 (49.0)	1.20 (1.14–1.27, *p* < 0.001)	1.15 (1.09–1.22, *p *< 0.001)
	Unknown	24 (0.3)	0.77 (0.50–1.20, *p* = 0.249)	1.03 (0.66–1.61, *p* = 0.887)
Distance (Crowfly) ***	11.2+ miles	3128 (41.9)	0.98 (0.93–1.04, *p* = 0.501)	1.00 (0.94–1.05, *p* = 0.863)
	<11.2 miles	3180 (42.6)	-	-
	Missing	1155 (15.5)	0.96 (0.89–1.03, *p* = 0.242)	1.19 (0.88–1.60, *p* = 0.252)
Treatment	SRS+Sys	416 (5.6)	-	-
	WBRT+Sys	3019 (40.5)	1.21 (1.08–1.36, *p* = 0.001)	1.19 (1.07–1.34, *p* = 0.002)
	Sys	2108 (28.2)	1.52 (1.36–1.71, *p* < 0.001)	1.51 (1.34–1.70, *p *< 0.001)
	SRS	51 (0.7)	2.62 (1.93–3.57, *p* < 0.001)	2.61 (1.91–3.55, *p* < 0.001)
	WBRT	620 (8.3)	4.08 (3.56–4.67, *p* < 0.001)	3.91 (3.41–4.47, *p* < 0.001)
	None	1249 (16.7)	5.45 (4.82–6.17, *p* < 0.001)	5.23 (4.63–5.92, *p* < 0.001)

Abbreviations: HR, hazard ratio; WBRT, whole-brain radiation therapy; SRS, stereotactic radiosurgery; Sys, systemic therapy; CI, confidence interval. *Median Income: Area-level median household income linked to patient ZIP codes using American Community Survey 2016–2020 data; analyzed as ≥$57,856 vs. <$57,856. ** Education: Percentage of adults aged ≥25 years within the patient’s ZIP code who did not complete high school; categorized as ≥9.1% vs. <9.1%. *** Distance (Crowfly): Straight-line (“crow-fly”) distance in miles between the centroid of the patient’s residential ZIP code and the treating facility; analyzed as <11.2 vs. ≥11.2 miles.

## Data Availability

The data supporting the findings of this study are available from the National Cancer Database (NCDB). Restrictions apply to the availability of these data, which were used under license for this study. Data are available from the American College of Surgeons and the American Cancer Society with permission.

## Supplementary Materials

The following supporting information can be downloaded at: https://www.mdpi.com/article/10.3390/cancers17233833/s1, Figure S1. Scaled Schoenfeld residual plots for covariates in the Cox proportional hazards model; Table S1. Accelerated failure time model for overall survival in patients with small cell lung cancer and brain metastases.

## Abbreviations

The following abbreviations are used in this manuscript:

BM	Brain metastases
CI	Confidence interval
CNS	Central nervous system
HR	Hazard ratio
IQR	Interquartile range
mOS	Median overall survival
NCDB	National Cancer Database
OS	Overall survival
PCI	Prophylactic cranial irradiation
SCLC	Small cell lung cancer
SRS	Stereotactic radiosurgery
Sys	Systemic therapy
WBRT	Whole-brain radiotherapy
