# Supplementary material for: Survival Determinants and Treatment Outcomes of Patients with Small Cell Lung Cancer and Brain Metastases: A U.S. National Analysis
Source: Cancers (Basel). 2025 Nov 29;17(23):3833. doi: 10.3390/cancers17233833 (PMC12691287; doi:10.3390/cancers17233833)
Supplement: Supplementary file 1 [file cancers-17-03833-s001.zip › cancers-3968229-supplementary.pdf]

Supplementary Figure S1. Scaled Schoenfeld residual plots for covariates in the Cox proportional hazards model.

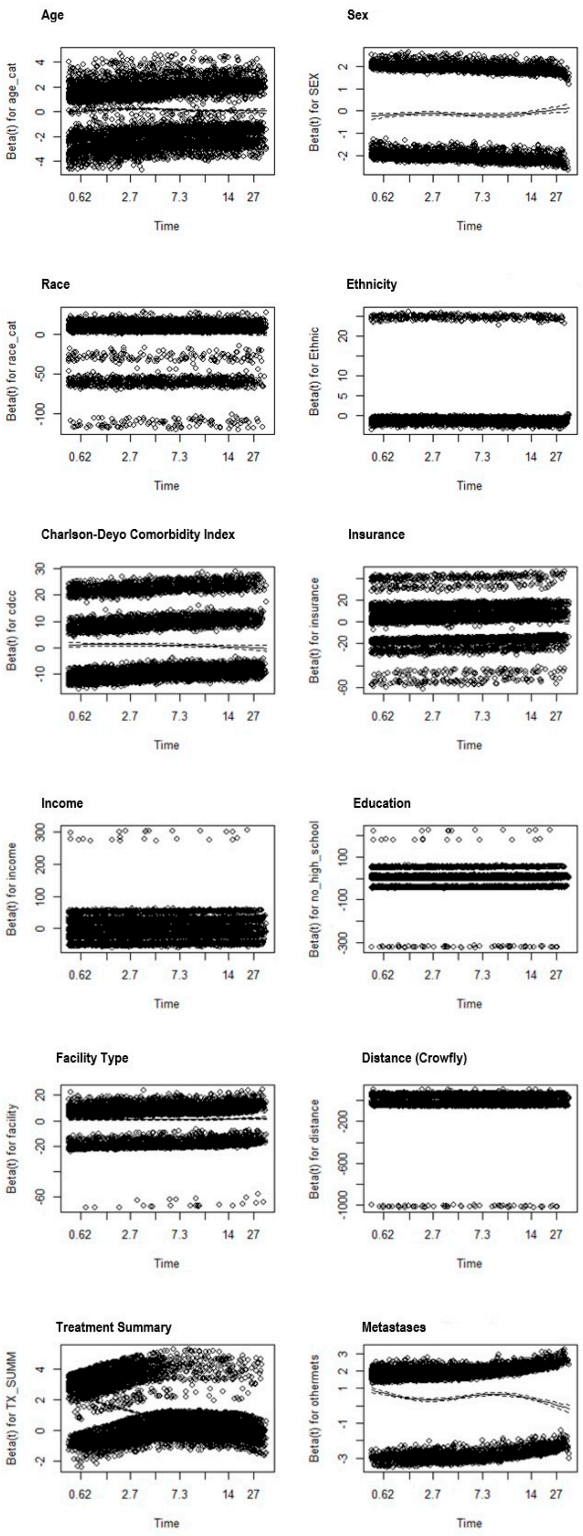

Supplementary Table S1. Accelerated Failure Time Model for Overall Survival in Patients with Small Cell Lung Cancer and Brain Metastases.

Variable	Estimate	SE	z	*p*-Value	Time Ratio
Age ≥65 years	−0.129	0.030	−4.3	<0.001	0.88
Female sex	0.149	0.022	6.8	<0.001	1.16
Black race	0.129	0.040	3.2	0.001	1.14
Asian race	0.232	0.100	2.3	0.020	1.26
Other race	0.058	0.089	0.7	0.512	1.06
Hispanic ethnicity	0.166	0.058	2.9	0.004	1.18
Comorbidity index = 1	−0.120	0.027	−4.5	<0.001	0.89
Comorbidity index = 2-3	−0.204	0.029	−7.0	<0.001	0.82
Medicare insurance	−0.147	0.033	−4.4	<0.001	0.86
Medicaid insurance	−0.146	0.040	−3.7	<0.001	0.86
Other insurance	0.135	0.077	1.7	0.081	1.14
Uninsured	−0.263	0.057	−4.6	<0.001	0.77
Income <$57,856	−0.079	0.028	−2.9	0.004	0.92
Income unknown	−0.332	0.216	−1.5	0.125	0.72
Education <9.1% (no HS)	−0.039	0.028	−1.4	0.163	0.96
Education unknown	0.454	0.254	1.8	0.074	1.57
Integrated network facility	−0.174	0.032	−5.5	<0.001	0.84
Community facility	−0.201	0.026	−7.8	<0.001	0.82
Facility unknown	0.349	0.218	1.6	0.109	1.42
Distance ≥11.2 miles	−0.007	0.024	−0.3	0.766	0.99
Distance missing	−0.150	0.137	−1.1	0.276	0.86
WBRT + Sys	−0.195	0.048	−4.0	<0.001	0.82
Sys only	−0.390	0.050	−7.8	<0.001	0.68
SRS only	−1.033	0.111	−9.3	<0.001	0.36
WBRT only	−1.388	0.057	−24.5	<0.001	0.25
No treatment	−1.610	0.053	−30.5	<0.001	0.20
Other metastases = Yes	−0.548	0.024	−22.9	<0.001	0.58
Log(scale)	0.052	0.008	6.9	<0.001	1.05

Abbreviations: AFT, accelerated failure time; SE, standard error; TR, time ratio; WBRT, whole-brain radiotherapy; SRS, stereotactic radiosurgery; Sys, systemic therapy.
